# First person – Timothée Gérard

**DOI:** 10.1242/bio.062589

**Published:** 2026-04-28

## Abstract

First Person is a series of interviews with the first authors of a selection of papers published in Biology Open, helping researchers promote themselves alongside their papers. Timothée Gérard is first author on ‘ Monitoring reproduction in cryptic small mammals: using body temperature to identify parturition in an endangered rodent’, published in BiO. Timothée conducted the research described in this article while a postdoc at the Hubert Curien Pluridisciplinary Institute in Dr Caroline Habold's lab in Strasbourg, France. He is now a postdoc at Hainan University in the lab of Prof. Zhibin Zhang, Haikou City, China, investigating the challenges of rodent management, conservation, and regulation in environments shaped by intense human activity.

**Figure:**
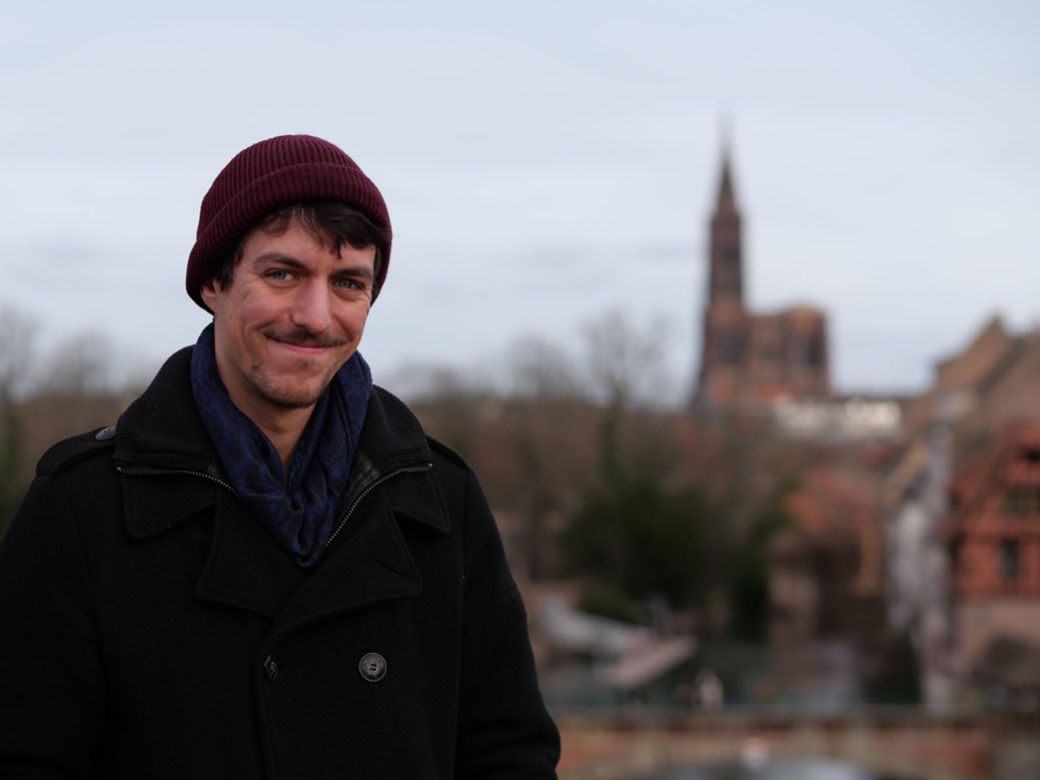
Timothée Gérard

**Describe your scientific journey and your current research focus**

During my studies, I quickly gravitated toward biology, driven by a long-standing interest in natural environments and landscapes. However, as I became more familiar with the challenges of biodiversity conservation, I realized that ‘nature’ cannot be treated as an isolated, self-contained entity to be managed independently. In human-modified environments, nature and human-activities are deeply intertwined, often generating tension between ecological, social, and economic priorities. Effective management policies therefore require a comprehensive approach that accounts for all of these factors.

My interest in this socio-ecosystem perspective was further developed during my PhD, which focused on reconciling the technical and economic constraints faced by French farmers with the conservation needs of an endangered farmland rodent, the common hamster (*Cricetus cricetus*). In seeking innovative and effective management solutions, I became particularly interested in the role of reproductive dynamics in shaping rodent populations. I now apply this perspective to the mitigation of zoonotic disease risks, investigating how fertility control methods may offer a more effective and sustainable alternative to conventional rodenticides for managing rat populations.

**Who or what inspired you to become a scientist?**

What inspired me to become a scientist was primarily the opportunity to develop and promote innovative, evidence-based solutions to societal challenges. This became especially clear during my PhD, through interactions with governmental policymakers and farmers. I found great fulfilment in my role as a scientist, being consulted on biodiversity needs while actively engaging in thinking about how to integrate ecological priorities with societal and economic considerations.

**How would you explain the main finding of your paper?**

In this paper, we demonstrate that the internal body temperature of female hamsters can serve as a reliable indicator of the timing of litter birth. Reproduction elevates body temperature throughout the reproductive period, from the first encounter with a male until the pups are weaned and leave the burrow. This pattern is particularly distinctive at the time of birth, when females exhibit a sharp, pronounced, and easily identifiable rise in temperature. The subsequent decline back to baseline is influenced by the number of pups being reared.

Our approach provides a valuable alternative, offering a more reliable way to monitor reproductive timing and enhancing future efforts to study and conserve hamster populations

**Figure:**
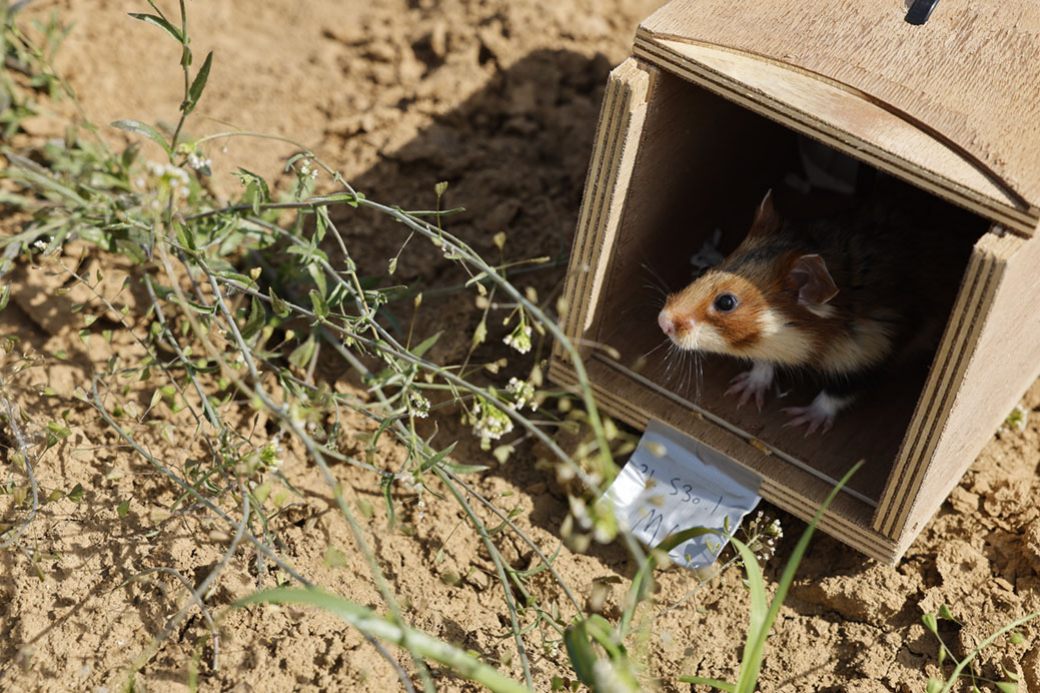
**A common hamster (*C. cricetus*) is being released for monitoring in a semi-natural environment.** The common hamster is an endangered rodent species that inhabits farmlands. They are intensively studied as part of the conservation efforts.

**What are the potential implications of this finding for your field of research?**

Studying the reproduction of wild endangered animals is essential for guiding and improving conservation efforts. However, this can be technically challenging for elusive species, such as the common hamster, which reproduces in burrows. Traditional methods for estimating litter birth dates are often imprecise or impractical, particularly in high-density populations. Our approach provides a valuable alternative, offering a more reliable way to monitor reproductive timing and enhancing future efforts to study and conserve hamster populations.

**Which part of this research project was the most rewarding?**

The elusive behaviour of wild hamsters makes them inherently mysterious. In the enclosure where we monitored them, trapping provided only brief glimpses of their activities. Accessing their body temperature data, therefore, was particularly rewarding, offering a window into aspects of their ‘intimate’ life. This proved valuable, not only for studying reproduction as described in the article, but also for understanding hibernation, where temperature measurements can reveal the timing of torpor and periods of activity, and it made me feel much closer to my study model.

**What's next for you?**

I am now beginning a new postdoctoral project at a university in China, focused on controlling rat populations in order to reduce zoonotic disease transmission risks. This project allows me to deepen my expertise in rodent management while addressing societal challenges. I am particularly interested in evaluating more ethical management alternatives – such as fertility control instead of poisons – while incorporating often-overlooked ecological principles, including the ecosystem services provided by rodents. This approach aligns with the One Health framework, connecting human health with rodent population stability and overall ecosystem health.
